# Supplementary material for: Generation of Lamprey Monoclonal Antibodies (Lampribodies) Using the Phage Display System
Source: Biomolecules. 2019 Dec 12;9(12):868. doi: 10.3390/biom9120868 (PMC6995607; doi:10.3390/biom9120868)
Supplement: Supplementary file 1 [file biomolecules-09-00868-s001.pdf]

biomolecules

MDPI

Supplementary Materials: Generation of lamprey monoclonal antibodies (lampribodies) using the phage display system.

Khan M.A. Hassan, John D. Hansen, Brantley R. Herrin, and Chris T. Amemiya

Contents

		Page
**Table S1**	Primer sequences used in this study	S2
**Table S2**	Analysis of the full length VLRB molecules found in the library from naïve sea lampreys	S3
**Figure S1**	Schematic presentation of the screening method of the naïve VLRB phage display library for lysozyme	S5
**Table S3**	ELISA result of the screening of the naïve VLRB phage display library for lysozyme	S6
**Figure S2**	Distribution of the Data in Table S3	S9
**Table S4**	ELISA positive clones from VLRB phage library from naïve sea lamprey with lysozyme bait	S10
**Figure S3**	ELISA test of the tentative competitor positive VLRB clones	S11
**Figure S5**	Schematic presentation of the anti-lysozyme VLRB:HEL spiking experiment	S12
**Table S5**	ELISA result of the screening of subtraction experiment	S13
**Figure S5**	Distribution of the Data in Table S5	S16
**Table S6**	Recovery percentage and category of the screened phages in the subtraction experiment	S17
**Figure S6**	Alignment of amino acid residues of the four full length VLRB.HEL molecules	S18
**Table S7**	Analysis of ELISA positive anti-IgM phages	S19
**Figure S7**	Alignment of the DNA and deduced proteins of the anti-IgM phages	S20

Table S1. Primer sequences used in this study

Serial	Primer name	5' to 3' sequence	No. of bases
1	PD-VLRB.F	TTACTCGCGGCCCAGCCGGCCATGGCGATGTGGATCAAGTGGATCGCCAC	50
2	PD-VLRB.R	CGCCTTGGCCTCCCGGGCCACGTTTCCTGCAGAGGGCGCAGGTCGA	46
3	CAPBSF1	ATGTGAGTTAGCTCACTCATTAGGC	25
4	GPIIICTR2	TGTCGTCTTTCCAGACGTTAGTAAATG	27
5	VLRB 5' UTR	CTCCGCTACTCGGCCTGCA	19
6	VLRB 3' UTR	CCGCCATCCCCGACCTTTG	19

Table S2. Analysis of the full length VLRB molecules found in the library from naïve sea lampreys. The variability of 27 full length VLRB molecules was examined for the usage of LRR cassettes. The putative protein sequences of LRR cassettes appearing after LRRNT to the beginning of the Connecting Peptide (CP) of each molecule were delineated and individual cassettes were numerically ordered, and their usage analyzed as follows. The number of LRR cassettes varied from 2 to 7 in these 27 molecules (shown as group I to VI). There was a preponderance of VLRB molecules with 3 (11 of 27) and 4 (7 of 27) LRR cassettes, together representing 66% of the sequenced molecules. The total number of LRR cassettes was 108 from the 27 VLRB sequences. Each of the cassettes was compared against the entire set to see if there was any repetitive usage. It was found that 74 cassettes were used only once, 14 were used twice and 2 were used thrice; therefore, there were 90 non-redundant cassettes in the entire set of 108. Each of these 90 cassettes was then compared against the non-redundant protein database (NCBI) using TBLASTN; 58 of the 90 cassettes completely matched sequences in the database (65%) and the rest were unique to the VLRB molecules analyzed. When similar searches were restricted to the sea lamprey (*Petromyzon marinus*) databases, only 30% of the cassettes (63/90) showed a match. The delineated LRR cassettes were numbered serially. The number in parenthesis indicates the appearance of the identical cassette in another molecule(s). NF: no perfect match was found in the NCBI blast search against the LRR cassette.

Serial	Clone ID	Total Amino acid	LRR1	LRR2	LRR3	LRR4	LRR5	LRR6	LRR7	Groups with equal number of LRRs	
										Group	Number of VLRBs
1	VLRB_02_05	253	1	2						I	1
2	VLRB_02_42	269	3 (27)	4 (NF)	5					II	11
3	VLRB_01_16	270	6 (15)	7	8						
4	VLRB_01_18	270	9	10 (70)	11 (NF)						
5	VLRB_01_20	270	12	13	14 (63)						
6	VLRB_01_24	270	15 (6)	16 (NF)	17						
7	VLRB_01_26	270	18	19 (NF)	20						
8	VLRB_02_41	270	21 (44)	22 (NF)	23						
9	VLRB_01_01	271	24	25 (65)	26						
10	VLRB_02_40	271	27 (3)	28 (NF)	29						
11	VLRB_02_18	273	30	31	32						
12	VLRB_01_25	277	33 (NF)	34 (NF)	35 (83)						

13	VLRB_01_15	293	36 (69)	37	38	39 (NF)				III	7
14	VLRB_01_17	294	40 (56)	41 (57)	42 (NF)	43 (55, 78)					
15	VLRB_02_23	294	44 (21)	45 (NF)	46 (NF)	47					
16	VLRB_02_34	294	48	49 (NF)	50	51 (NF)					
17	VLRB_02_37	294	52 (90, 96)	53	54	55 (43, 78)					
18	VLRB_02_17	295	56 (40)	57 (41)	58	59					
19	VLRB_02_31	297	60	61	62	63 (14)					
20	VLRB_01_14	317	64	65 (25)	66	67 (NF)	68 (NF)			IV	4
21	VLRB_01_11	318	69 (36)	70 (10)	71	72 (NF)	73 (NF)				
22	VLRB_01_03	319	74	75 (NF)	76	77 (NF)	78 (43, 55)				
23	VLRB_02_36	322	79	80 (NF)	81 (NF)	82	83 (35)				
24	VLRB_01_13	337	84	85 (NF)	86	87 (NF)	88	89		V	3
25	VLRB_01_08	343	90 (52, 96)	91 (97)	92 (98)	93 (99)	94 (100)	95 (NF)			
26	VLRB_01_09	343	96 (52, 90)	97 (91)	98 (92)	99 (93)	100 (94)	101 (NF)			
27	VLRB_01_06	366	102 (NF)	103 (NF)	104 (NF)	105 (NF)	106 (NF)	107	108 (NF)	VI	1

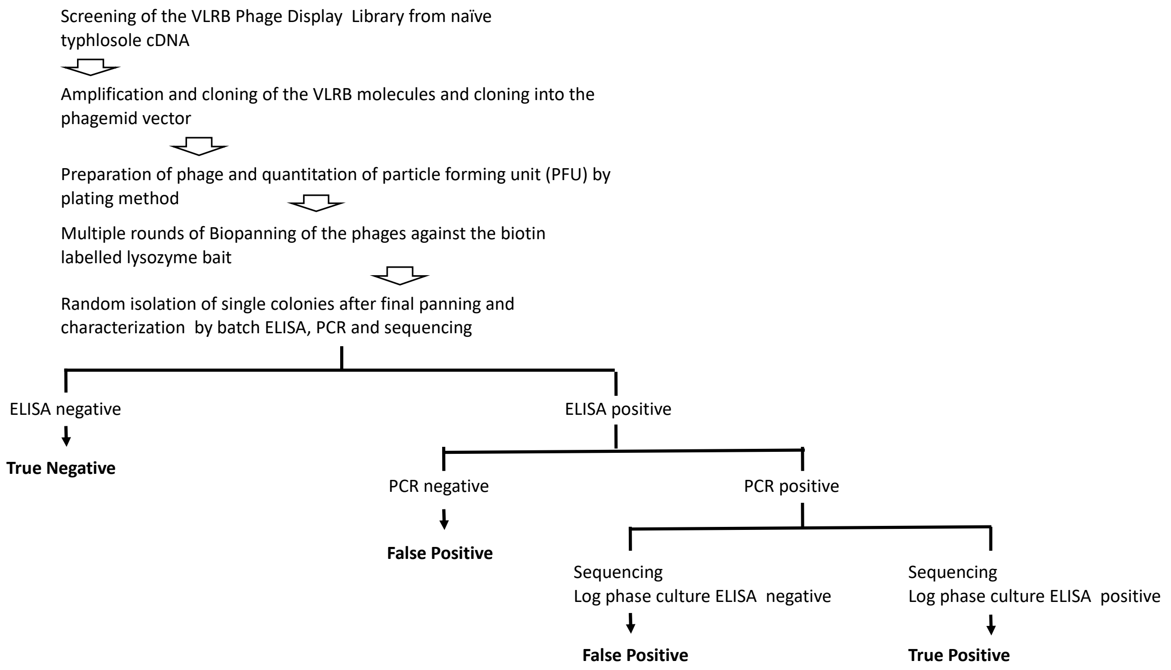

Figure S1. Schematic presentation of the screening method of the VLRB phage display library from the typhlosole of unimmunized animals by biopanning for lysozyme. The VLRB phage display library was constructed and used for multiple round of biopanning for lysozyme. After final round of panning, individual phage clones were characterized by batch ELISA to eliminate true negative clones. ELISA positive clones were further examined by PCR for insert size; phagemid with no insert or short insert was called PCR negative and that with insert, PCR positive. The clone was then sequenced, and ELISA was repeated with phage prepared from log phase culture after titer determination.

Table S3A. ELISA result of the screening of the naïve VLRB phage display library for lysozyme. After 2nd biopanning of the VLRB phage library from naïve sea lamprey with lysozyme bait, 48 randomly isolated phagemid clones were examined by ELISA with biotinylated lysozyme coated plates, PCR and sequencing. PCR sizes are estimates from the agarose gel electrophoresis. The clones were sorted for estimated insert size of ascending order.

Serial	Phages	Estimated PCR size (Kb)	ELISA (OD)	Phage status	Insert Verification PCR/Sequence
1	2P7_2	0.40	0.063	TN	PCR
2	2P7_3	0.40	0.057	TN	PCR
3	2P7_4	0.40	0.060	TN	PCR
4	2P7_6	0.40	0.057	TN	PCR
5	2P7_8	0.40	0.056	TN	PCR
6	2P7_11	0.40	0.065	TN	PCR
7	2P7_14	0.40	0.060	TN	PCR
8	2P7_17	0.40	0.057	TN	PCR
9	2P7_18	0.40	0.110	FP	PCR
10	2P7_19	0.40	0.061	TN	PCR
11	2P7_21	0.40	0.059	TN	PCR
12	2P7_23	0.40	0.054	TN	PCR
13	2P7_24	0.40	0.071	TN	PCR
14	2P7_25	0.40	0.266	FP	PCR
15	2P7_27	0.40	0.409	FP	PCR
16	2P7_28	0.40	0.061	TN	PCR
17	2P7_30	0.40	0.108	FP	PCR
18	2P7_33	0.40	0.061	TN	PCR
19	2P7_36	0.40	0.060	TN	PCR
20	2P7_45	0.40	0.532	TN	PCR
21	2P7_16	0.50	0.073	TN	PCR
22	2P7_39	0.50	0.064	TN	PCR
23	2P7_40	0.50	0.099	TN	PCR
24	2P7_42	0.50	0.055	TN	PCR
25	2P7_47	0.50	0.159	FP	PCR
26	2P7_38	0.60	2.106	FP	Sequence
27	2P7_48	0.60	0.059	TN	PCR
28	2P7_1	0.80	0.095	TN	PCR
29	2P7_5	0.80	0.061	TN	PCR
30	2P7_10	0.80	0.053	TN	PCR
31	2P7_15	0.80	0.064	TN	PCR
32	2P7_20	0.80	0.059	TN	PCR

33	2P7_22	0.80	0.059	TN	PCR
34	2P7_31	0.80	0.303	FP	Sequence
35	2P7_35	0.80	0.079	TN	PCR
36	2P7_37	0.80	0.056	TN	PCR
37	2P7_41	0.80	0.052	TN	PCR
38	2P7_46	0.80	0.422	FP	Sequence
39	2P7_7	0.90	0.082	TN	PCR
40	2P7_12	0.90	0.054	TN	PCR
41	2P7_9	1.00	0.057	TN	PCR
42	2P7_13	1.00	0.054	TN	PCR
43	2P7_26	1.00	0.054	TN	PCR
44	2P7_32	1.00	0.071	TN	PCR
45	2P7_34	1.00	0.058	TN	PCR
46	2P7_43	1.00	0.074	TN	PCR
47	2P7_29	1.10	0.053	TN	PCR
48	2P7_44	1.10	0.074	TN	PCR

Table S3B. Duplicate experiment as in table S3A

Serial	Phages	Estimated PCR size (Kb)	ELISA (OD)	Phage clone status	Insert Verification PCR/Sequence
1	2P8_1	0.4	0.087	TN	PCR
2	2P8_2	0.4	0.056	TN	PCR
3	2P8_4	0.4	0.078	TN	PCR
4	2P8_6	0.4	0.077	TN	PCR
5	2P8_7	0.4	0.066	TN	PCR
6	2P8_8	0.4	0.056	TN	PCR
7	2P8_11	0.4	0.094	TN	PCR
8	2P8_14	0.4	0.099	TN	PCR
9	2P8_15	0.4	0.078	TN	PCR
10	2P8_16	0.4	0.067	TN	PCR
11	2P8_17	0.4	0.07	TN	PCR
12	2P8_18	0.4	0.059	TN	PCR
13	2P8_19	0.4	0.056	TN	PCR
14	2P8_21	0.4	0.059	TN	PCR
15	2P8_24	0.4	0.082	TN	PCR
16	2P8_27	0.4	0.074	TN	PCR
17	2P8_29	0.4	0.059	TN	PCR
18	2P8_30	0.4	0.091	TN	PCR
19	2P8_31	0.4	0.133	FP	PCR

20	2P8_34	0.4	0.063	TN	PCR
21	2P8_35	0.4	0.07	TN	PCR
22	2P8_36	0.4	0.058	TN	PCR
23	2P8_38	0.4	0.05	TN	PCR
24	2P8_40	0.4	0.059	TN	PCR
25	2P8_41	0.4	0.062	TN	PCR
26	2P8_42	0.4	0.052	TN	PCR
27	2P8_43	0.4	0.051	TN	PCR
28	2P8_44	0.4	0.086	TN	PCR
29	2P8_45	0.4	0.517	FP	PCR
30	2P8_48	0.4	0.494	FP	PCR
31	2P8_37	0.5	0.097	TN	PCR
32	2P8_10	0.7	0.055	TN	PCR
33	2P8_33	0.7	2.467	FP	Sequence
34	2P8_3	0.8	0.056	TN	PCR
35	2P8_5	0.8	0.062	TN	PCR
36	2P8_9	0.8	0.058	TN	PCR
37	2P8_12	0.8	0.059	TN	PCR
38	2P8_13	0.8	0.073	TN	PCR
39	2P8_20	0.8	0.053	TN	PCR
40	2P8_26	0.8	0.069	TN	PCR
41	2P8_28	0.8	0.057	TN	PCR
42	2P8_32	0.8	0.058	TN	PCR
43	2P8_39	0.8	0.055	TN	PCR
44	2P8_46	0.8	0.042	TN	PCR
45	2P8_47	0.8	0.039	TN	PCR
46	2P8_22	0.9	0.053	TN	PCR
47	2P8_23	0.9	0.062	TN	PCR
48	2P8_25	0.9	0.052	TN	PCR

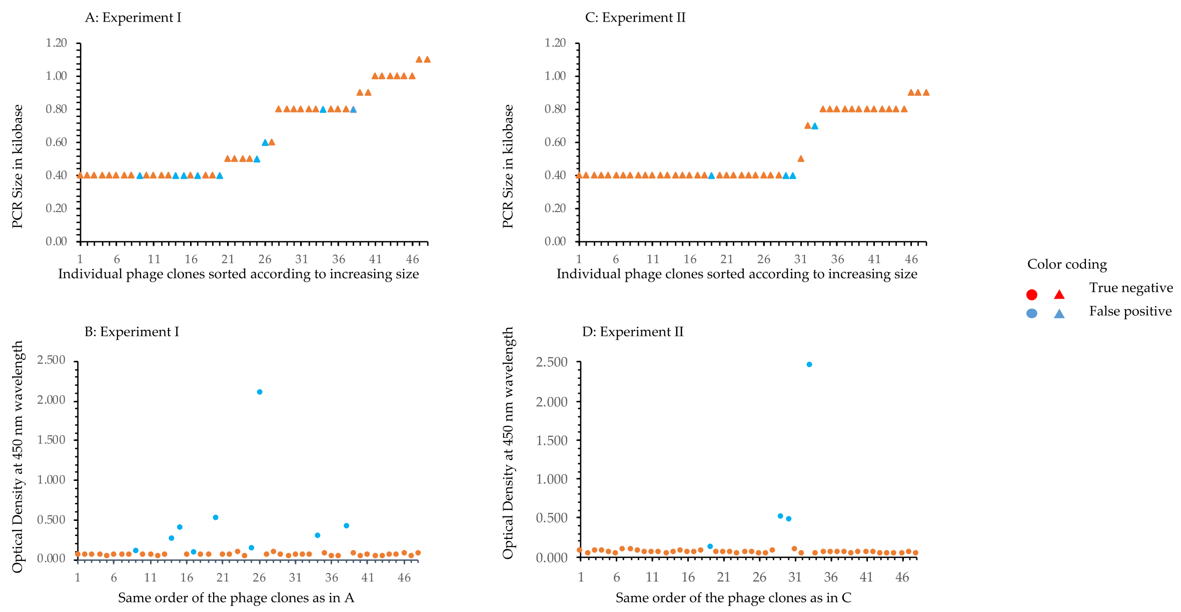

Figure S2. Distribution of the insert size of the phage clones examined and the corresponding ELISA signals after 2nd panning of the naïve VLRB library with lysozyme bait. In the first panning, 10^9 PFU of the library were incubated with biotinylated hen egg lysozyme on ELISA plates. The bound phages were recovered, amplified and used for second panning. The recovery percentage of the trapped phages after the second panning was measured by plating and was found to be 0.0003% and 0.0017% in duplicate experiments, suggesting that there were no authentic lysozyme binding VLRBs in the library. The single colonies thus obtained after the second panning were further characterized for their binding affinity for lysozyme following a simple scheme (described in Figure S2). 48 randomly selected single colonies from each of the duplicate experiments were grown in batch in order to: 1) prepare phage for ELISA; 2) extract phagemid DNA to examine the insert by PCR and sequencing as needed. The initial screening of phages was done by ELISA (OD) readings above 0.1 were considered positive). ELISA negative phages were categorized as true negative and not characterized any further. ELISA positive phages were further characterized for VLRB insert by PCR using primers CAPBSF1 and GPIICTR2R1 (Figure S1, Table S1), located upstream and downstream of the *Bgl*I cloning site respectively (the phagemid without any insert produced a fragment of 329 bp). Phages showing no insert or very short insert were called PCR negative and categorized as false positives. Phages having large inserts were sequenced to determine if they were VLRB molecules. All the phages examined in an experiment were first sorted for their estimated PCR product size (and sequence data) of the insert as shown in (A) and (C), listed in ascending order (Table S3) and plotted with the corresponding OD reading in ELISA for each clone examined. For experiment I, (A) shows the order of the phage clones arranged in order of increasing size (filled triangle), and (B) shows the optical density reading at 450 nm (filled circle) of the same clones in the same order. The same was done for experiment II as in (B) and (D). Inserts with estimated sizes of 0.4 kb were phages with either no insert or small inserts. Color coding for the clones: red triangle and circle are true negatives, blue triangles and circles, false positives.

Table S4. ELISA positive clones from VLRB phage library from naïve sea lamprey with lysozyme bait along with OD at screening. Insert sized were determined by sequencing.

Serial	Phage clones	Estimated PCR size (Kb)	ELISA (OD)	Phage clone status	Insert Verification Sequence (bp)
1	2P7_31	0.8	0.303	FP	471*
2	2P7_38	0.6	2.106	FP	438**
3	2P7_46	0.8	0.422	FP	807
4	2P8_33	0.7	2.467	FP	438**

* This clone is truncated at the CP after 4 residues

** These clones are identical and have a stop codon after the first 4 amino acids.

FP: false positive

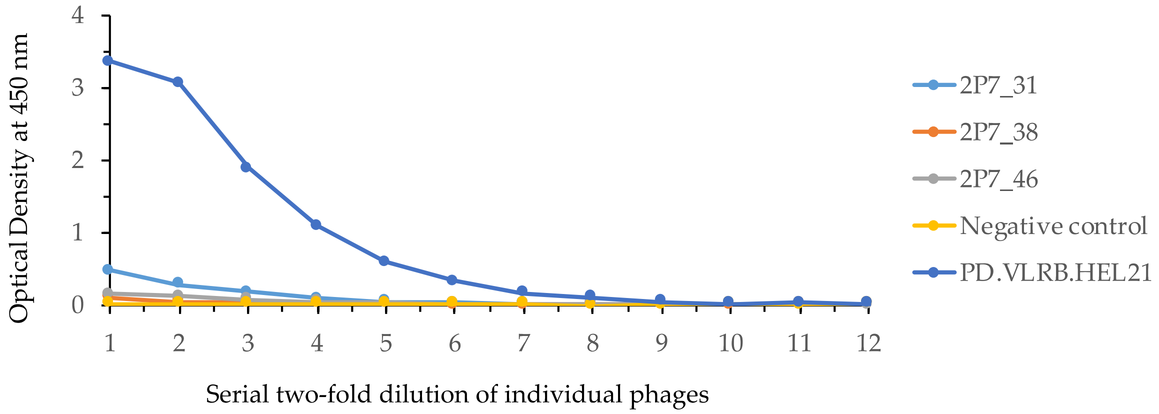

Figure S3. ELISA test of the tentative competitor positive VLRB clones. In duplicate experiments (Figure 2B and 2D), 83.33% and 91.67% of the clones were negative for ELISA (true negative). In one of the duplicate experiments (Figure 2B, Table S3A), of the 8 ELISA positive phages, 5 had either no insert or short inserts (2P7_18, 2P7_25, 2P7_27, 2P7_30 and 2P7_45), therefore those were considered false positives. In the other experiment (Figure 2D, Table S3B), 3 of the 4 ELISA positive phages had either no insert or short inserts (2P8_31, 2P8_45 and 2P8_48), therefore those were also considered false positives. The rest of the 4 clones from two experiments with larger inserts were sequenced (Table S4). Two of these (2P7_38 and 2P8_33), one from each of the duplicate experiments, were identical and had a G to A transition at the 14th base starting after the first Adenine. In the deduced protein sequence, the G to A transition introduced an amber stop codon after the first four amino acids. It also showed truncation after 37 residues of LRRCT. The amber mutation was within the primer sequence; therefore, it was difficult to ascertain if it represented a true VLRB transcript. TG1 used for phage packaging is an amber codon suppressor strain and therefore, allowed formation of phages with this clone. Another (2P7_31) was truncated after 4 residues of the connecting peptide. Only one phagemid clone was full length VLRB (2P7_46) with 3 LRR cassettes in the variable region. Fresh phages were prepared from three tentative competitor positive phagemids, 2P7_31, 2P7_38 and 2P7_46, and ELISA was done on wells coated with biotinylated lysozyme with two-fold serial dilution starting from $1X10^9$ PFU pADL10b-1-8 and VLRB.HEL21 phages were used as positive and negative controls respectively. Numbers in X-axis denotes serial half dilution of the phage solutions from $1{\times}10^9$ to 4. $\times10^6$ PFU.

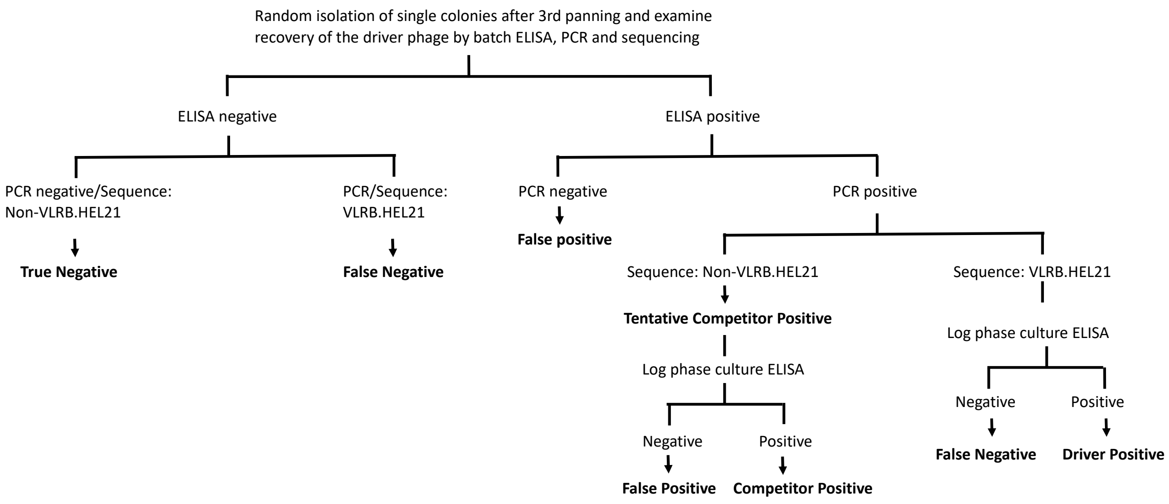

Figure S4. Schematic presentation of the screening method of the driver VLRB.HEL21 phage in the subtraction experiment. In duplicate experiments, 1 PFU of VLRB.HEL21 phage was mixed with 10^9 PFU of the library and the mixture was applied to biotinylated lysozyme coated wells of the ELISA plates for panning. Trapped phages were recovered and used for amplification as described above. As only one PFU of the driver phage was used, the titer determination and estimation of the recovery of the trapped phage steps were omitted after 1st panning to allow generation of phages. After amplification, a small aliquot of the phage solution was used for a 2nd round of panning. After elution of the bound phages and infection of the TG1 cells, a small aliquot of the cells was plated to estimate the recovery of the trapped phage. The recovery of the trapped phages was to 0.014% and 0.0115% respectively for each of the duplicate experiments. The improvement of the recovery of the trapped phage in the subtraction experiments compared to that of the library alone (0.0003% and 0.0017%) could be attributed to the amplification of the driver phage. Panning was repeated for a 3rd time and single colonies were obtained for further screening. Phages were used for ELISA and the phagemids for VLRB insert analysis by PCR and sequencing. The phages were categorized into groups of true negative, false positive, competitor positive, false negative and driver positive. The final verification of a competitor positive and a driver positive phage was performed by sequencing of the insert and ELISA of the phage prepared from log phase culture for lysozyme binding. False positive, and false negative categories were examined by sequencing and ELISA at two different steps of the screening for accurate characterization.

Table S5A. ELISA result of the screening of subtraction experiment. After 3rd panning for subtraction, 48 randomly isolated phagemid clones were examined by ELIZA with biotinylated lysozyme coated plates, PCR and sequencing. PCR sizes are estimates from the agarose gel electrophoresis and were used for sorting in the ascending order.

Serial	Phages	Estimated PCR size (Kb)	ELISA (OD)	Phage status	Insert check: PCR/ PCR Digest/Sequence
1	3P1_3	0.4	0.414	FP	PCR
2	3P1_5	0.4	0.756	FP	PCR
3	3P1_7	0.4	0.829	FP	PCR
4	3P1_9	0.4	0.798	FP	PCR
5	3P1_10	0.4	1.17	FP	PCR
6	3P1_12	0.4	0.737	FP	PCR
7	3P1_14	0.4	2.66	FP	PCR
8	3P1_17	0.4	0.406	FP	PCR
9	3P1_19	0.4	0.825	FP	PCR
10	3P1_20	0.4	0.334	FP	PCR
11	3P1_21	0.4	0.905	FP	PCR
12	3P1_24	0.4	0.362	FP	PCR
13	3P1_25	0.4	0.354	FP	PCR
14	3P1_29	0.4	0.637	FP	PCR
15	3P1_32	0.4	1.047	FP	PCR
16	3P1_34	0.4	0.618	FP	PCR
17	3P1_35	0.4	0.34	FP	PCR
18	3P1_36	0.4	0.323	FP	PCR
19	3P1_37	0.4	0.987	FP	PCR
20	3P1_38	0.4	0.542	FP	PCR
21	3P1_40	0.4	0.774	FP	PCR
22	3P1_41	0.4	0.779	FP	PCR
23	3P1_42	0.4	0.339	FP	PCR
24	3P1_43	0.4	0.676	FP	PCR
25	3P1_44	0.4	0.653	FP	PCR
26	3P1_45	0.4	0.181	FP	PCR
27	3P1_46	0.4	0.871	FP	PCR
28	3P1_15	0.7	0.363	TCP	PCR
29	3P1_33	0.7	0.078	TN	PCR
30	3P1_6	0.9	2.173	TCP	PCR
31	3P1_13	0.9	0.369	TCP	PCR
32	3P1_18	0.9	2.179	TCP	PCR
33	3P1_27	0.9	0.572	TCP	PCR

34	3P1_30	1.1	0.099	TN	PCR Digest
35	3P1_1	1.2	0.536	DP	PCR Digest/Sequence
36	3P1_2	1.2	2.876	DP	PCR Digest/Sequence
37	3P1_4	1.2	2.62	DP	PCR Digest/Sequence
38	3P1_8	1.2	2.671	DP	PCR Digest/Sequence
39	3P1_11	1.2	2.655	DP	PCR Digest/Sequence
40	3P1_16	1.2	2.601	DP	PCR Digest/Sequence
41	3P1_22	1.2	2.654	DP	PCR Digest/Sequence
42	3P1_23	1.2	2.641	DP	PCR Digest/Sequence
43	3P1_26	1.2	2.614	DP	PCR Digest/Sequence
44	3P1_28	1.2	2.607	DP	PCR Digest/Sequence
45	3P1_31	1.2	2.609	DP	PCR Digest/Sequence
46	3P1_39	1.2	2.616	DP	PCR Digest/Sequence
47	3P1_47	1.2	2.623	DP	PCR Digest/Sequence
48	3P1_48	1.2	2.581	DP	PCR Digest/Sequence

Kb: kibase, OD: Optical density, TN: ture negative, DP: Driver positive, TCP: Tentative competitor positive, FP: false positive, FN: false negative

Table S5B. Duplicate experiment as in table S5A

Serial	Phages	Estimated PCR size (Kb)	ELISA (OD)	Phage status	Insert check: PCR/ PCR Digest/Sequence
1	3P2_1	0.4	0.936	FP	PCR
2	3P2_4	0.4	0.577	FP	PCR
3	3P2_5	0.4	0.624	FP	PCR
4	3P2_6	0.4	0.483	FP	PCR
5	3P2_8	0.4	0.072	TN	PCR
6	3P2_9	0.4	0.708	FP	PCR
7	3P2_10	0.4	0.164	FP	PCR
8	3P2_11	0.4	0.119	FP	PCR
9	3P2_12	0.4	0.814	FP	PCR
10	3P2_13	0.4	1.207	FP	PCR
11	3P2_14	0.4	1.153	FP	PCR
12	3P2_15	0.4	0.547	FP	PCR
13	3P2_16	0.4	0.433	FP	PCR
14	3P2_17	0.4	0.289	FP	PCR
15	3P2_18	0.4	0.53	FP	PCR
16	3P2_19	0.4	0.429	FP	PCR
17	3P2_21	0.4	0.697	FP	PCR
18	3P2_22	0.4	0.555	FP	PCR

19	3P2_23	0.4	0.722	FP	PCR
20	3P2_24	0.4	0.584	FP	PCR
21	3P2_25	0.4	0.221	FP	PCR
22	3P2_26	0.4	1.135	FP	PCR
23	3P2_28	0.4	0.964	FP	PCR
24	3P2_29	0.4	1.082	FP	PCR
25	3P2_32	0.4	2.54	FP	PCR
26	3P2_33	0.4	1.192	FP	PCR
27	3P2_34	0.4	0.265	FP	PCR
28	3P2_38	0.4	0.103	FP	PCR
29	3P2_39	0.4	0.288	FP	PCR
30	3P2_40	0.4	2.469	FP	PCR
31	3P2_41	0.4	1.11	FP	PCR
32	3P2_43	0.4	1.103	FP	PCR
33	3P2_44	0.4	1.399	FP	PCR
34	3P2_45	0.4	0.171	FP	PCR
35	3P2_46	0.4	0.176	FP	PCR
36	3P2_47	0.4	0.832	FP	PCR
37	3P2_37	0.6	0.134	TCP	PCR
38	3P2_30	0.7	2.321	TCP	PCR
39	3P2_31	0.7	0.905	TCP	PCR
40	3P2_35	0.7	2.004	TCP	PCR
41	3P2_36	0.7	0.062	TN	PCR
42	3P2_48	0.7	0.245	TCP	PCR
43	3P2_20	0.9	0.548	TCP	PCR
44	3P2_42	1.1	0.053	TN	PCR Digest
45	3P2_2	1.2	2.589	DP	PCR Digest/Sequence
46	3P2_3	1.2	2.705	DP	PCR Digest/Sequence
47	3P2_7	1.2	2.689	DP	PCR Digest/Sequence
48	3P2_27	1.2	2.572	DP	PCR Digest/Sequence

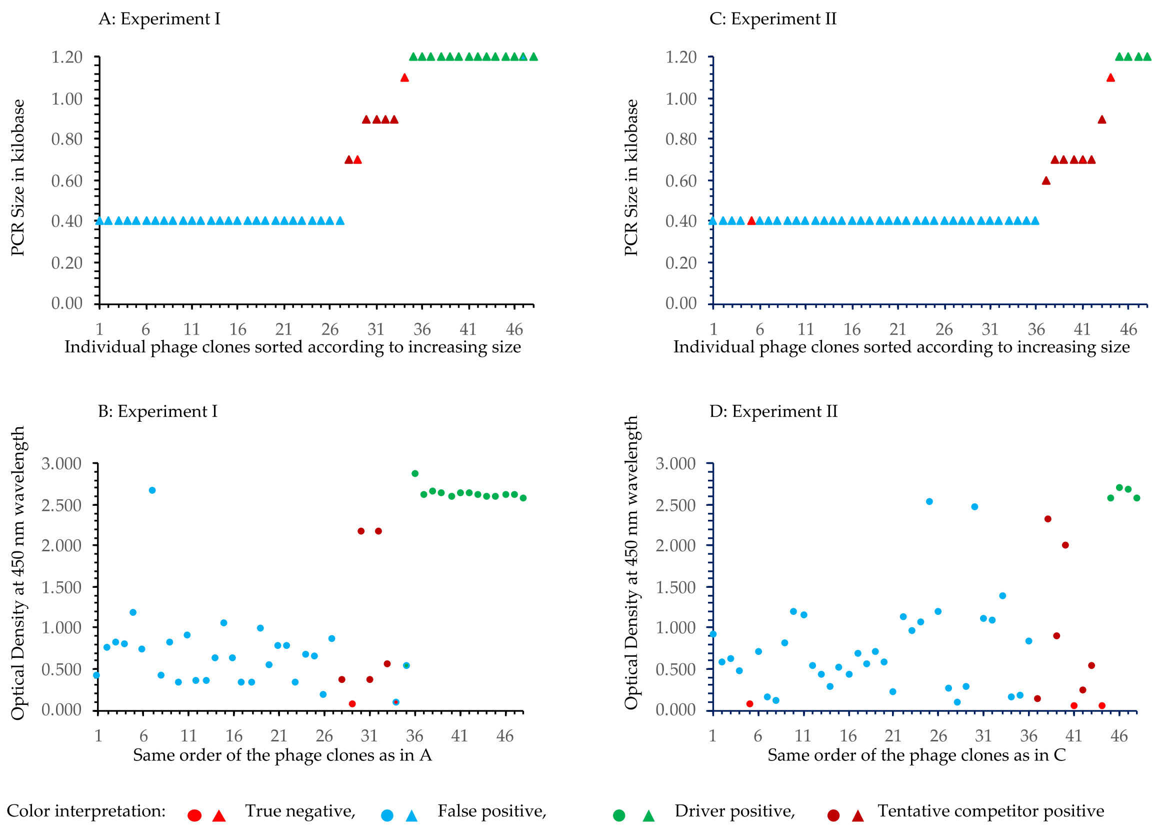

Figure S5. Insert size and ELISA signal intensity plots of the candidate phage clones of the subtraction experiment after 3rd panning. A total of 48 randomly isolated clones from each of the duplicate experiments were examined by ELISA and the corresponding insert sizes were determined by PCR, enzyme digestion and sequencing. The clones were first sorted for size and plotted against OD readings in ELISA from each clone as in figure 2. Filled triangles are estimated PCR sizes and filled circles are the OD readings of the corresponding clones. A and B are for experiment I and C and D are for experiment II. Inserts with estimated sizes of 0.4 kb were phages with either no insert or small inserts. Color coding for the clones: red triangle and circle, true negatives; and blue triangles and circles, false positives; green triangle and circle, driver positive; brown triangle and circle, tentative competitor positive.

Table S6. Recovery percentage and category of the randomly screed phages in subtraction experiments. 3P1 and 3P2 are two duplicate experiments.

Phage status	Experiment I (3P1)		Experiment II (3P2)	
	Number	Percent	Number	Percent
True negative (TN)	2	4.17	3	6.25
False negative (FN)	0	0.00	0	0.00
False positive (FP)	27	56.25	35	72.92
Tentative Competitor positive (TCP)	5	10.42	6	12.50
Dirver positive (DP)	14	29.17	4	8.33

```
             Signal Peptide------->LRRNT------------------------>LRR1----
VLRB.HEL.1   MWIKWIATLVAFGALVQSAVAACPSQCSCDQTTVYCHNRRLTSVPAGIPTDRQNLWLYDN  60
VLRB.HEL.2D  MWIKWIATLVAFGALVQSAVAACPSQCSCSGTTVDCSGKSLASVPTGIPTTTQVLYLYDN  60
VLRB.HEL.21  MWIKWIATLVAFGALVQSAVAACPSQCSCDQTTVYCHSRRLTSVPAGIPTTTRVLHLHTN  60
10bLysA1P2_1 MWIKWIATLVAFGALVQSAV-ACPSQCSCSGTEVNCAGKSLASVPAGIPTTTRVLYLNSN  59

             ---------->LRR2------------------->LRR3------------------->LRR4
VLRB.HEL.1   QITKLEPGVFDRLTKLTHLSLGYNQLKSVPRGAFD------------------------- 105
VLRB.HEL.2D  QITKLEPGVFDRLTQLTRLDLDNNQLTVLPAGVFDKLTQLTQLSLNDNQLKSIPRGAFD- 119
VLRB.HEL.21  QITKLEPGVFDSLTQLTELYLSANQLTTLPAGLFDRLVKLKELWLNNNQLTSLPTGVFDK 120
10bLysA1P2_1 QITKLEPGVFDRLGNLQRLWLNNNQLTALPAGVFDKLTLLAGLSLDQNQLKSIPRGTFD- 119

             ---------------------->CP------------>LRRCT------------------
VLRB.HEL.1   -----------------------NLKSLTHIWLLNNPWDCECSDILYLKNWIVQHASIVN 142
VLRB.HEL.2D  -----------------------NLKSLTHIWLLNNPWDCACSDILYLSRWISQHPGLVF 156
VLRB.HEL.21  LTQLTQLGLWDNQLKSIPRGAFDNLKSLTHIYLFNNPWDCECSDILYLKNWIVQHASIVN 180
10bLysA1P2_1 -----------------------NLKSLTHIYLFNNPWDCECSDILYLKNWIVQHASIVN 156

             --------------------------------->Stalk-------------------
VLRB.HEL.1   PGNYGGVDNVKCFGTNTPVRAVTEASTSPSKCPGYVATTTTPTTTTPEFIPETTTSPQPV 202
VLRB.HEL.2D  GYLNLDPDSARCSGTNTPVRAVTEASTSPSKCPGYVATTTTPTTTTPEFIPETTTSPQPV 216
VLRB.HEL.21  PGN-GGVDNVKCSGTNTPVRAVTEASTSPSKCPGYVATTTTPTTTTPEFIPETTTSPQPV 239
10bLysA1P2_1 PGN-GGVDNVKCSGTNTPVRAVTEASTSPSKCPGYVATTTTPTTTTPEFIPETTTSPQPV 215

             -------------------------------------------------------
VLRB.HEL.1   ITTQKPKPLWNFNCTSIQERKNDGGDCGKPACTTLLNCANFLSCLCSTCALCRKR 257
VLRB.HEL.2D  ITTQKPKPLWNFNCTSIQERKNDGGDCGKPACTTLLNCANFLSCLCSTCALCRKR 271
VLRB.HEL.21  ITTQKPKPLWNFNCTSIQERKNDGGDCGKPACTTLLNCANFLSCLCSTCALCRKR 294
10bLysA1P2_1 ITTQKPKPLWNFNCTSIQERKNDGGDCGKPACTTLLNCANFLSCLCSTCALCRKR 270
```

Figure S6. Alignment of the VLRB.HEL clones. Alignment of amino acid residues of the three full length VLRB.HEL synthesized with the diversity regions isolated previously (9) and the one isolated lysozyme by phage display screening. The structural motifs corresponding to the amino acid sequences are shown above the alignment. Identical amino acids are shaded.

Table S7. Sequencing of 10 ELISA positive anti-IgM phages. One (10bIgM3P_2) was 810 bp, the rest were 807 bp in length. In these 10 clones, 4 and 10 were identical and called Clone A; 6, 7, 8 and 9 were identical, called Clone B; 1, 3 and 5 were non clonal.

Serial	Phages	VLRB insert length (bp)	Clonality
1	10bIgM3P_4	807	clone A
2	10bIgM3P_10	807	clone A
3	10bIgM3P_6	807	clone B
4	10bIgM3P_7	807	clone B
5	10bIgM3P_8	807	clone B
6	10bIgM3P_9	807	clone B
7	10bIgM3P_1	807	Non clonal
8	10bIgM3P_3	807	Non clonal
9	10bIgM3P_5	807	Non clonal
10	10bIgM3P_2	810	Non clonal

```
                Signal Peptide------------------------------------------->LRRNT
10bIgM3P_4      ATGTGGATCAAGTGGATCGCCACGCTGGTCGCCTTTGGCGCCCTGGTGCAAAGTGCGGTA  60
10bIgM3P_6      ATGTGGATCAAGTGGATCGCCACGCTGGTCGCCTTTGGCGCCCTGGTGCAAAGTGCGGTA  60
10bIgM3P_1      ATGTGGATCAAGTGGATCGCCACGCTGGTCGCCTTTGGCGCCCTGGTGCAAAGTGCGGTA  60
10bIgM3P_3      ATGTGGATCAAGTGGATCGCCACGCTGGTCGCCTTTGGCGCCCTGGTGCAAAGTGCGGTA  60
10bIgM3P_5      ATGTGGATCAAGTGGATCGCCACGCTGGTCGCCTTTGGCGCCCTGGTGCAAAGTGCGGTA  60
10bIgM3P_2      ATGTGGATCAAGTGGATCGCCACGCTGGTCGCCTTTGGCGCCCTGGTGCAAAGTGCGGTA  60

                ------------------------------------------------------------
10bIgM3P_4      GCATGTCCCTCGCAGTGTTCGTGCAGAGGGACATATGTGGACTGTGATAGCAGAAGCCTC 120
10bIgM3P_6      GCATGTCCCTCGCAGTGTTCGTGCAGAGGGACATATGTGGACTGTGATAGCAGAAGCCTC 120
10bIgM3P_1      GCATGTCCCTCGCAGTGTTCGTGCAGAGGGACATATGTGGACTGTGATAGCAGAAGCCTC 120
10bIgM3P_3      GCATGTCCCTCGCAGTGTTCGTGCAGAGGGACATATGTGGACTGTGATAGCAGAAGCCTC 120
10bIgM3P_5      GCATGTCCCTCGCAGTGTTCGTGCAGAGGGACATATGTGGACTGTGATAGCAGAAGCCTC 120
10bIgM3P_2      GCATGTCCCTCGCAGTGTTCGTGCAGAGGGACATATGTGGACTGTGATAGCAGAAGCCTC 120

                --------------------------->LRR1----------------------------
10bIgM3P_4      GCGTCTGTGCCTGCGGGAATCCCTACCACCACGCAAGTGCTGTATTTGTACAGCAATCAA 180
10bIgM3P_6      GCGTCTGTGCCTGCGGGAATCCCTACCACCACGCAAGTGCTGTATTTGTACAGCAATCAA 180
10bIgM3P_1      GCGTCTGTGCCTGCGGGAATCCCTACCACCACGCAAGTGCTGTATTTGTACAGCAATCAA 180
10bIgM3P_3      GCGTCTGTGCCTGCGGGAATCCCTACCACCACGCAAGTGCTGTATTTGTACAGCAATCAA 180
10bIgM3P_5      GCGTCTGTGCCTGCGGGAATCCCTACCACCACGCAAGTGCTGTATTTGTACAGCAATCAA 180
10bIgM3P_2      GCGTCTGTGCCTGCGGGAATCCCTACCACCACGCAAGTGCTGTATTTGTACAGCAATCAA 180

                ----------------------------->LRR2--------------------------
10bIgM3P_4      ATCACGAAGCTCGAGCCAGGGGTGTTTGATAGTCTGGCGAATTTGAGGGAGCTTCATCTG 240
10bIgM3P_6      ATCACGAAGCTCGAGCCAGGGGTGTTTGATAGTCTGGCGAATTTGAGGGAGCTTCATCTG 240
10bIgM3P_1      ATCACGAAGCTCGAGCCAGGGGTGTTTGATAGTCTGGCGAATTTGAGGGAGCTTCATCTG 240
10bIgM3P_3      ATCACGAAGCTCGAGCCAGGGGTGTTTGATAGTCTGGCGAATTTGAGGGAGCTTCATCTG 240
10bIgM3P_5      ATCACGAAGCTCGAGCCAGGGGTGTTTGATAGTCTGGCGAATTTGAGGGAGCTTCATCTG 240
10bIgM3P_2      ATCACGAAGCTCGAGCCAGGGGTGTTTGATAGTCTGGCGAATTTGAGGGAGCTTCATCTG 240

                ------------------------------------------->LRR3------------
10bIgM3P_4      TGGGGGAACAAGCTGGTGTCTCTTCCCCCTGGTGTGTTTGACCGGCTGGGGAAGCTGCAG 300
10bIgM3P_6      TGGGGGAACAAGCTGGTGTCTCTTCCCCCTGGTGTGTTTGACCGGCTGGGGAAGCTGCAG 300
10bIgM3P_1      TGGGGGAACAAGCTGGTGTCTCTTCCCCCTGGTGTGTTTGACCGGCTGGGGAAGCTGCAG 300
10bIgM3P_3      TGGGGGAACAAGCTGGTGTCTCTTCCCCCTGGTGTGTTTGACCGGCTGGGGAAGCTGCAG 300
10bIgM3P_5      TGGGGGAACAAGCTGGTGTCTCTTCCCCCTGGTGTGTTTGACCGGCTGGGGAAGCTGCAG 300
10bIgM3P_2      TGGGGGAACAAGCTGGTGTCTCTTCCCCCTGGTGTGTTTGACCGGCTGGGGAAGCTGCAG 300

                ------------------------------------------------------>CP----
10bIgM3P_4      CATTTAGATCTGTCAAAGAACCAGCTGAAGAGCATTCCCAGGGGCGCCTTTGACAACCTC 360
```

```
10bIgM3P_6    CATTTAGATCTGTCAAAGAACCAGCTGAAGAGCATTCCCAGGGGCGCCTTTGACAACCTC 360
10bIgM3P_1    CATTTAGATCTGTCAAAGAACCAGCTGAAGAGCATTCCCAGGGGCGCCTTTGACAACCTC 360
10bIgM3P_3    CATTTAGATCTGTCAAAGAACCAGCTGAAGAGCATTCCCAGGGGCGCCTTTGACAACCTC 360
10bIgM3P_5    CATTTAGATCTGTCAAAGAACCAGCTGAAGAGCATTCCCAGGGGCGCCTTTGACAACCTC 360
10bIgM3P_2    CATTTAGATCTGTCAAAGAACCAGCTGAAGAGCATTCCCAGGGGCGCCTTTGACAACCTC 360

              ----------------------------------->LRRCT--------------------
10bIgM3P_4    AAGAGCCTCACTCACATCTATCTGTTCAACAACCCCTGGGACTGCGAGTGTTCGGACATC 420
10bIgM3P_6    AAGAGCCTCACTCAGATCTGGCTGTACAACAACCCCTGGGACTGCGAGTGTTCGGACATC 420
10bIgM3P_1    AAGAGCCTCACTCAGATCTGGCTGTACAACAACCCCTGGGACTGCGAGTGTTCGGACATC 420
10bIgM3P_3    AAGAGCCTCACTCACATCTATCTGTTCAACAACCCCTGGGACTGCGAGTGTTCGGACATC 420
10bIgM3P_5    AAGAGCCTCACTCAGATCTGGCTGTACAACAACCCCTGGGACTGCGAGTGTTCGGACATC 420
10bIgM3P_2    AAGAGCCTCACTCACATCTATCTGTTCAACAACCCCTGGGACTGCGAGTGTTCGGACATC 420

              ------------------------------------------------------------
10bIgM3P_4    CTCTATCTGAAGAACTGGATTGTGCAGCACGCAAGCATCGTGAATCCA---GGCAACGGG 477
10bIgM3P_6    CTCTATCTGAAGAACTGGATTGTGCAGCACGCAAGCATCGTGAATCCA---GGCAACGGG 477
10bIgM3P_1    CTCTATCTGAAGAACTGGATTGTGCAGCACGCAAGCATCGTGAATCCA---GGCAACGGG 477
10bIgM3P_3    CTCTATCTGAAGAACTGGATTGTGCAGCACGCAAGCATCGTGAATCCA---GGCAACGGG 477
10bIgM3P_5    CTCTATCTGAAGAACTGGATTGTGCAGCACGCAAGCATCGTGAATCCA---GGCAACGGG 477
10bIgM3P_2    CTCTATCTGAAGAACTGGATTGTGCAGCACGCAAGCATCGTGAATCCATCGGGCTATGGG 480

              ------------------------------>Stalk------------------------
10bIgM3P_4    GGAGTTGATAACGTGAAGTGCTCTGGTACCAATACCCCCGTCCGTGCGGTCACCGAGGCC 537
10bIgM3P_6    GGAGTTGATAACGTGAAGTGCTCTGGTACCAATACCCCCGTCCGTGCGGTCACCGAGGCC 537
10bIgM3P_1    GGAGTTGATAACGTGAAGTGCTCTGGTACCAATACCCCCGTCCGTGCGGTCACCGAGGCC 537
10bIgM3P_3    GGAGTTGATAACGTGAAGTGCTCTGGTACCAATACCCCCGTCCGTGCGGTCACCGAGGCC 537
10bIgM3P_5    GGAGTTGATAACGTGAAGTGCTCTGGTACCAATACCCCCGTCCGTGCGGTCACCGAGGCC 537
10bIgM3P_2    GGAGTTGATAACGTGAAGTGCTCTGGTACCAATACCCCCGTCCGTGCGGTCACCGAGGCC 540

              ------------------------------------------------------------
10bIgM3P_4    AGCACTAGCCCCTCGAAATGCCCAGGCTACGTTGCTACGACCACGACGCCGACGACGACC 597
10bIgM3P_6    AGCACTAGCCCCTCGAAATGCCCAGGCTACGTTGCTACGACCACGACGCCGACGACGACC 597
10bIgM3P_1    AGCACTAGCCCCTCGAAATGCCCAGGCTACGTTGCTACGACCACGACGCCGACGACGACC 597
10bIgM3P_3    AGCACTAGCCCCTCGAAATGCCCAGGCTACGTTGCTACGACCACGACGCCGACGACGACC 597
10bIgM3P_5    AGCACTAGCCCCTCGAAATGCCCAGGCTACGTTGCTACGACCACGACGCCGACGACGACC 597
10bIgM3P_2    AGCACTAGCCCCTCGAAATGCCCAGGCTACGTTGCTACGACCACGACGCCGACGACGACC 600

              ------------------------------------------------------------
10bIgM3P_4    ACGCCCGAATTCATCCCTGAGACCACCACCTCGCCGCAGCCCGTGATCACAACCCAGAAA 657
10bIgM3P_6    ACGCCCGAATTCATCCCTGAGACCACCACCTCGCCGCAGCCCGTGATCACAACCCAGAAA 657
10bIgM3P_1    ACGCCCGAATTCATCCCTGAGACCACCACCTCGCCGCAGCCCGTGATCACAACCCAGAAA 657
```

```
10bIgM3P_3    ACGCCCGAATTCATCCCTGAGACCACCACCTCGCCGCAGCCCGTGATCACAACCCAGAAA 657
10bIgM3P_5    ACGCCCGAATTCATCCCTGAGACCACCACCTCGCCGCAGCCCGTGATCACAACCCAGAAA 657
10bIgM3P_2    ACGCCCGAATTCATCCCTGAGACCACCACCTCGCCGCAGCCCGTGATCACAACCCAGAAA 660

              ------------------------------------------------------------
10bIgM3P_4    CCCAAGCCTCTGTGGAATTTCAACTGCACCTCAATTCAGGAGAGGAAGAACGACGGTGGC 717
10bIgM3P_6    CCCAAGCCTCTGTGGAATTTCAACTGCACCTCAATTCAGGAGAGGAAGAACGACGGTGGC 717
10bIgM3P_1    CCCAAGCCTCTGTGGAATTTCAACTGCACCTCAATTCAGGAGAGGAAGAACGACGGTGGC 717
10bIgM3P_3    CCCAAGCCTCTGTGGAATTTCAACTGCACCTCAATTCAGGAGAGGAAGAACGACGGTGGC 717
10bIgM3P_5    CCCAAGCCTCTGTGGAATTTCAACTGCACCTCAATTCAGGAGAGGAAGAACGACGGTGGC 717
10bIgM3P_2    CCCAAGCCTCTGTGGAATTTCAACTGCACCTCAATTCAGGAGAGGAAGAACGACGGTGGC 720

              ------------------------------------------------------------
10bIgM3P_4    GACTGCGGAAAGCCCGCCTGCACAACTCTCCTGAACTGCGCGAATTTCCTCAGCTGCCGC 777
10bIgM3P_6    GACTGCGGAAAGCCCGCCTGCACAACTCTCCTGAACTGCGCGAATTTCCTCAGCTGCCTC 777
10bIgM3P_1    GACTGCGGAAAGCCCGCCTGCACAACTCTCCTGAACTGCGCGAATTTCCTCAGCTGCCCC 777
10bIgM3P_3    GACTGCGGAAAGCCCGCCTGCACAACTCTCCTGAACTGCGCGAATTTCCTCAGCTGCCTC 777
10bIgM3P_5    GACTGCGGAAAGCCCGCCTGCACAACTCTCCTGAACTGCGCGAATTCCCTCAGCTGCCTC 777
10bIgM3P_2    GACTGCGGAAAGCCCGCCTGCACAACTCTCCTGAACTGCGCGAATTTCCTCAGCTGCCTC 780

              ------------------------------
10bIgM3P_4    TACTCGACCTGCGCCCTCTGCAGGAAACGT 807
10bIgM3P_6    TGCTCGACCTGCGCCCTCTGCAGGAAACGT 807
10bIgM3P_1    TGCTCGACCTGCGCCCTCTGCAGGAAACGT 807
10bIgM3P_3    TGCTCGACCTGCGCCCTCTGCAGGAAACGT 807
10bIgM3P_5    TGCTCGACCTGCGCCCTCTGCAGGAAACGT 807
10bIgM3P_2    TGCTCGACCTGCGCCCTCTGCAGGAAACGT 810
```

Figure S7A. Alignment of the DNA sequences of the anti-human IgM VLRBs identified by phage display screening. The structural motifs represented by the DNA sequences are shown above the alignment.

```
              Signal Peptide---->LRRNT-------------------------->LRR1-----
10bIgM3P_4    MWIKWIATLVAFGALVQSAVACPSQCSCRGTYVDCDSRSLASVPAGIPTTTQVLYLYSNQ
10bIgM3P_6    MWIKWIATLVAFGALVQSAVACPSQCSCRGTYVDCDSRSLASVPAGIPTTTQVLYLYSNQ
10bIgM3P_1    MWIKWIATLVAFGALVQSAVACPSQCSCRGTYVDCDSRSLASVPAGIPTTTQVLYLYSNQ
10bIgM3P_3    MWIKWIATLVAFGALVQSAVACPSQCSCRGTYVDCDSRSLASVPAGIPTTTQVLYLYSNQ
10bIgM3P_5    MWIKWIATLVAFGALVQSAVACPSQCSCRGTYVDCDSRSLASVPAGIPTTTQVLYLYSNQ
10bIgM3P_2    MWIKWIATLVAFGALVQSAVACPSQCSCRGTYVDCDSRSLASVPAGIPTTTQVLYLYSNQ
              ************************************************************

              -------->LRR2------------------->LRR3------------------->CP-
```

```
10bIgM3P_4    ITKLEPGVFDSLANLRELHLWGNKLVSLPPGVFDRLGKLQHLDLSKNQLKSIPRGAFDNL
10bIgM3P_6    ITKLEPGVFDSLANLRELHLWGNKLVSLPPGVFDRLGKLQHLDLSKNQLKSIPRGAFDNL
10bIgM3P_1    ITKLEPGVFDSLANLRELHLWGNKLVSLPPGVFDRLGKLQHLDLSKNQLKSIPRGAFDNL
10bIgM3P_3    ITKLEPGVFDSLANLRELHLWGNKLVSLPPGVFDRLGKLQHLDLSKNQLKSIPRGAFDNL
10bIgM3P_5    ITKLEPGVFDSLANLRELHLWGNKLVSLPPGVFDRLGKLQHLDLSKNQLKSIPRGAFDNL
10bIgM3P_2    ITKLEPGVFDSLANLRELHLWGNKLVSLPPGVFDRLGKLQHLDLSKNQLKSIPRGAFDNL
              ************************************************************

              ----------->LRRCT-------------------------------------------
10bIgM3P_4    KSLTHIYLFNNPWDCECSDILYLKNWIVQHASIVNP-GNGGVDNVKCSGTNTPVRAVTEA
10bIgM3P_6    KSLTQIWLYNNPWDCECSDILYLKNWIVQHASIVNP-GNGGVDNVKCSGTNTPVRAVTEA
10bIgM3P_1    KSLTQIWLYNNPWDCECSDILYLKNWIVQHASIVNP-GNGGVDNVKCSGTNTPVRAVTEA
10bIgM3P_3    KSLTHIYLFNNPWDCECSDILYLKNWIVQHASIVNP-GNGGVDNVKCSGTNTPVRAVTEA
10bIgM3P_5    KSLTQIWLYNNPWDCECSDILYLKNWIVQHASIVNP-GNGGVDNVKCSGTNTPVRAVTEA
10bIgM3P_2    KSLTHIYLFNNPWDCECSDILYLKNWIVQHASIVNPSGYGGVDNVKCSGTNTPVRAVTEA
              ****:*:*:*************************** *  ********************

              ------->Stalk-----------------------------------------------
10bIgM3P_4    STSPSKCPGYVATTTTPTTTTPEFIPETTTSPQPVITTQKPKPLWNFNCTSIQERKNDGG
10bIgM3P_6    STSPSKCPGYVATTTTPTTTTPEFIPETTTSPQPVITTQKPKPLWNFNCTSIQERKNDGG
10bIgM3P_1    STSPSKCPGYVATTTTPTTTTPEFIPETTTSPQPVITTQKPKPLWNFNCTSIQERKNDGG
10bIgM3P_3    STSPSKCPGYVATTTTPTTTTPEFIPETTTSPQPVITTQKPKPLWNFNCTSIQERKNDGG
10bIgM3P_5    STSPSKCPGYVATTTTPTTTTPEFIPETTTSPQPVITTQKPKPLWNFNCTSIQERKNDGG
10bIgM3P_2    STSPSKCPGYVATTTTPTTTTPEFIPETTTSPQPVITTQKPKPLWNFNCTSIQERKNDGG
              ************************************************************

              ------------------------------
10bIgM3P_4    DCGKPACTTLLNCANFLSCRYSTCALCRKR
10bIgM3P_6    DCGKPACTTLLNCANFLSCLCSTCALCRKR
10bIgM3P_1    DCGKPACTTLLNCANFLSCPCSTCALCRKR
10bIgM3P_3    DCGKPACTTLLNCANFLSCLCSTCALCRKR
10bIgM3P_5    DCGKPACTTLLNCANSLSCLCSTCALCRKR
10bIgM3P_2    DCGKPACTTLLNCANFLSCLCSTCALCRKR
              *************** ***  *********
```

Figure S7B. Alignment of the deduced amino acid sequences of the anti-human IgM VLRBs identified by phage display screening. The structural motifs represented by the DNA sequences are shown above the alignment.
